# Time to death and risk factors among tuberculosis patients in Northern Ethiopia

**DOI:** 10.1186/s13104-018-3806-7

**Published:** 2018-10-04

**Authors:** Solomon Weldegebreal Asgedom, Daniel Tesfaye, Yirga Legesse Nirayo, Tesfay Mehari Atey

**Affiliations:** School of Pharmacy, College of Health Sciences, Mekelle University, Mekelle, Ethiopia

**Keywords:** Time to death, Tuberculosis, Risk factors, Ethiopia

## Abstract

**Objective:**

The main objective of this study was to assess time to death and associated risk factors among tuberculosis (TB) patients.

**Results:**

A total of 769 TB patients were studied and of those, 87 (11.3%) patients died. All of the deaths occurred within 7 months of anti-tuberculosis therapy. Extra-pulmonary TB (AHR = 17.376, 95% CI; 3.88–77.86, p < 0.001) as compared to pulmonary TB and cotrimoxazole prophylaxis therapy (CPT) (AHR = 0.15, 95% CI; 0.03–0.74, p = 0.02) were found to be the predictors of mortality. We noticed higher rates of mortality. Extra-pulmonary TB patients have high risk and TB-HIV co-infected patients who received CPT have low risk of death. Improving early diagnosis of extra-pulmonary TB and early CPT initiation of TB-HIV co-infected patients could minimize patient’s mortality.

**Electronic supplementary material:**

The online version of this article (10.1186/s13104-018-3806-7) contains supplementary material, which is available to authorized users.

## Introduction

Worldwide, mortality and incidence rates due to tuberculosis (TB) and new incidences of TB cases were continued to fall in 2016 globally [1]. Despite the decrease, TB remains the ninth 10 causes of death and the leading cause from a single infectious agent ranking above HIV/AIDS [1, 2]. In 2016, there were an estimated 10.4 million new (incident) TB cases and 1.3 million TB deaths worldwide, in which about half and a quarter of the incident cases occurred i. in the WHO South East Asia region and the WHO Africa region. The global TB death estimation in 2016 was 1.3 million. The number of TB deaths fell by 20% in TB incidents and 35% in TB deaths compared with levels in 2015 [2].

Ethiopia is one of the 30 high TB burden countries. Moreover, Ethiopia is also one of the 14 high TB, Multidrug resistance tuberculosis (MDR-TB) and TB/HIV coinfection burden countries [2]. TB is still one of the leading causes of mortality in Ethiopia as well as worldwide [3]. According to the 2017 WHO report, 153,119 cases of MDR-TB and Rifampicin resistant (RR-TB) were notified in 2016 worldwide. In Ethiopia the estimated incidence of MDR-TB/RR-TB in 2016 was 2.7% [2].

To improve treatment outcome of TB patients and hence to achieve country wise milestone goals, understanding the attributable factors for TB mortality and identifying mortality determinants remain indispensable to find out strategies and interventions to solve the problem. Accordingly, deterioration in TB healthcare services and increase in MDR and XRD resistances were facilitators of mortality due to TB [4, 5]. On the other hand, antiretroviral therapy (ART), cotrimoxazole preventive therapy (CPT), and type of TB diagnosis were found to be predictors of TB mortality [6]. Thus risk of death can be possibly reduced through understanding of mortality predictors and by improving patient care [7, 8]. Studying patient’s survival status and identifying the risk factors for mortality help health care professionals to plan effective interventions to reduce death rates. In the study settings time to death and predictors of mortality were not yet studied. Therefore, the main objective of this study was to assess time to death and associated risk factors among TB patients in Mekelle Hospital (MH) and Ayder Comprehensive Specialized Hospital (ACSH), Northern Ethiopia.

## Main text

### Methods

Institutions based cross-sectional study was carried out in two hospitals of the region that is, MH and ACSH, Mekelle, Northern Ethiopia. Directly Observed Therapy Short course (DOTS) clinic is employed in the whole country. Hence both hospitals are operating DOTS. In the country, DOTS is implementing under the National Tuberculosis and Leprosy Control Program (NTLCP) of Ethiopia. DOTS is employed in many of the country through appropriate TB diagnosis. Therefore, patients are diagnosed of tuberculosis via sputum smear investigation for the presence of Acid Fast bacilli, chest radiography and pathological examination such as biopsy and fine needle aspiration cytology (FNAC).

All TB patients who received DOTS therapy at TB clinics of ACSH and Mekelle Hospital was the source patients and all TB patients who registered from 2012 to 2016 at TB clinics of both hospitals who fulfilled the inclusion criteria was the study population. TB patients who transferred out, patients with incomplete medical records, patients with unknown/unrecorded outcome and patients who received DOTS therapy before 2012 were excluded from the study. Medical records of the patients were retrospectively followed starting from the day of anti-TB therapy initiation until the time of the outcome (death). Survival time in days was measured from the date of initiation of anti-tuberculosis until the time of death or the end of follow up. TB patients who died were assumed as failures and patients who remained alive at the end of the treatment were considered as censored. All patients who fulfil the inclusion criteria were consecutively recruited. Hence, a total of 769 patient record cards of TB patients who started anti-TB chemotherapy from 2012 up to 2016 were included and retrospectively followed.

Survival time was the primary out come and sex, place of residence, age, baseline weight, HIV status, ART, CPT, type of TB, TB category at presentation and year of anti-TB therapy were the independent variables studied.

#### Data collection tool and procedure

Ethical clearance and approval of the study was obtained from Mekelle University Ethical review board, College of Health Sciences before starting the data collection. Subsequent permission was granted from ACSH and Mekelle hospital to access data. Patients medical chart was entirely confidential and private information like name and address were protected. A data collection format was developed using standardized TB entry and follow up form employed by the TB clinic was utilized to extract the required data from patients’ medical records. The data was collected by reviewing registers, follow up form and patients’ card. To ensure quality of data collection process two pharmacists were recruited and trained for 2 days. Pre-test was conducted on 39 patients before the actual data collection to assess the data collection tool. The pre tested patients were not included in the analysis. Based on the pretest finding, amendments and arrangements was made on the data collection tool. Data collection was closely supervised by the principal investigator.

The following operational definitions were used in this study: an overall mortality was defined if a patient who dies for any reason during the course of treatment. The ‘time to death’ or ‘survival time’ was measured in days from the date of initiation of anti-tuberculosis to the time of overall mortality.

#### Statistical analysis

All completed data was entered in SPSS version 21 and examined for completeness and consistency during data analysis. The data was entered and cleaned by a data clerk and the principal investigator before analysis. The survival time was measured in days calculated using the time interval between the date of anti-TB initiation and the date of death or censoring. The Kaplan–Meier model was used to estimate the survival probability after anti-TB initiation. Log Rank (Mantel-Cox), Breslow (Generalized Wilcoxon) and Tarone-W were used to compare survival curves. The Cox proportional hazard model was used to identify predictors of mortality. Level of significance was declared at p level less than 0.05.

### Results

#### Demographic and clinical characteristics of patients

A total of 1012 patients were involved in the study. From the total TB patients recruited, 115 patients transferred out, 12 dropped cases, 111 patients had incomplete medical records and 5 patients were lost from follow up. Finally, a total of 769 TB patients were enrolled and analyzed in the study from both hospitals. Among the total patients studied, 445 (57.9%) patients were males. The average mean age of the patients was found to be 35.1 ± 16.9 ranged from 1 to 83 years old. From the total patients studied 203 (26.4%) patients were 25–34 years old. Majority [667 (86.7%)] of the patients enrolled in the study were urban residents. Regarding HIV status of the patients, 474 (61.6%) patients were HIV negative (non-reactive). In addition, concerning TB category at presentation, 707 (91.9%) patients were new TB patients (Table 1).

**Table 1:** Socio-demographic characteristics of TB cases (n = 769) in Northern Ethiopia, 2012–2016

Variable	Type of TB			All TB cases (%)
	SPPTB (%)	SNPTB (%)	EPTB (%)	
Gender				
Male	80 (18.0)	120 (27.0)	245 (55.1)	445 (57.9)
Female	42 (13.0)	79 (24.4)	203 (62.7)	324 (42.1)
Age (mean ± SD) in years [35.1 ± 16.9]				
≤ 14	0 (0.0)	4 (9.3)	39 (90.7)	43 (5.6)
15–24	33 (20.4)	42 (25.9)	87 (53.7)	162 (21.1)
25–34	53 (26.1)	37 (18.2)	113 (55.7)	203 (26.4)
35–44	26 (14.4)	43 (23.8)	112 (61.9)	181 (23.5)
45–54	3 (4.3)	28 (40.6)	38 (55.1)	69 (9.0)
55–64	0 (0.0)	11 (35.3)	20 (64.5)	31 (4.0)
≥ 65	7 (8.8)	34 (42.5)	39 (48.8)	80 (10.4)
Residence				
Rural	9 (8.8)	17 (16.7)	76 (74.5)	102 (13.3)
Urban	113 (16.9)	182 (27.3)	372 (55.8)	667 (86.7)
HIV status				
Non-reactive	82 (17.3)	141 (29.7)	251 (53.0)	474 (61.6)
Reactive	40 (13.6)	58 (19.7)	197 (66.8)	295 (38.4)
Year of treatment				
2012	4 (7.3)	25 (45.5)	26 (47.3)	55 (7.2)
2013	76 (19.9)	101 (26.5)	204 (53.5)	381 (49.5)
2014	31 (17.1)	34 (18.8)	116 (64.1)	181 (23.5)
2015	8 (7.0)	32 (28.1)	74 (64.9)	114 (14.8)
2016	3 (7.9)	7 (18.4)	28 (73.7)	38 (4.9)
Body weight at initial stage of treatment				
≤ 14	0 (0.0)	2 (5.9)	32 (94.1)	34 (4.4)
15–34	3 (8.3)	21 (58.3)	12 (33.3)	36 (4.7)
35–44	38 (19.0)	59 (29.5)	103 (51.5)	200 (26.0)
45–54	57 (16.3)	78 (22.3)	215 (61.4)	350 (45.5)
≥ 55	24 (16.1)	39 (26.2)	86 (57.7)	149 (19.4)
CPT				
No	11 (15.1)	18 (24.7)	44 (60.3)	73 (9.5)
Yes	29 (12.9)	44 (19.6)	152 (67.6)	225 (29.3)
Not candidate	82 (17.4)	137 (29.1)	252 (53.5)	471 (61.2)
ART				
Yes	29 (15.0)	37 (19.2)	127 (65.8)	193 (25.1)
No	9 (11.4)	21 (25.6)	52 (63.4)	82 (10.7)
Non-reactive	84 (17.1)	141 (28.7)	266 (54.2)	494 (64.2)
Patients category				
New	108 (15.3)	178 (25.2)	421 (59.5)	707 (91.9)
Relapse/failure/default	8 (100.0)	0 (0.0)	0 (0.0)	8 (1.0)
Transfer in	6 (31.6)	9 (47.4)	4 (21.1)	19 (2.5)
Others	0 (0.0)	12 (34.3)	23 (65.7)	35 (4.6)

*ART* antiretroviral therapy, *CPT* cotrimoxazole prophylaxis therapy, *EPTB* extra-pulmonary tuberculosis, *HIV* human immune deficiency virus, *SPPTB* smear positive pulmonary tuberculosis, *SNPTB* smear negative pulmonary tuberculosis, *SD* standard deviation

**Fig. 1:**
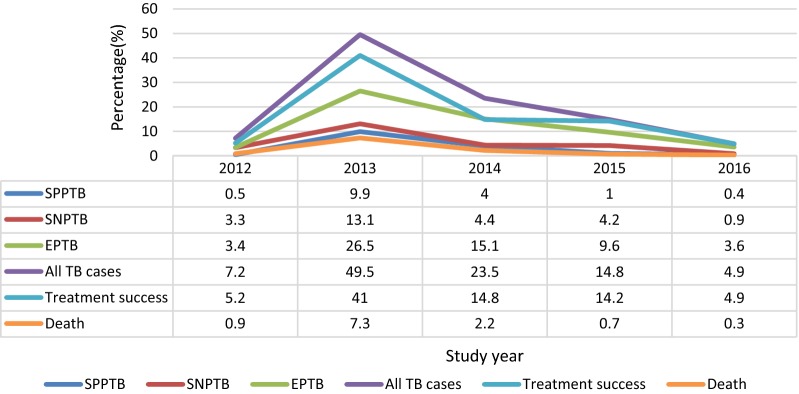
Treatment outcome and mortality among TB patients in ACSH and MH in 2012–2016. *SPPTB* smear positive pulmonary tuberculosis, *SNPTB* smear negative pulmonary tuberculosis, *EPTB* extra-pulmonary tuberculosis

The median time of the TB patients to death was 10 days and the average mean (± SD) time to death was 25.8 ± 44.9 days. The crude mortality rate per 100 persons years observation (PYO) was 11.3/100 (11.3%) per annum. As shown in Fig. 1, mortality due to TB reached a peak in 2013 and then significantly decreased thereafter. The proportion of death from smear positive pulmonary TB, smear negative pulmonary TB and extra pulmonary TB patients were 1.3%, 1.4%, and 8.6% respectively.

All (100%) death cases of the patient occurred within less than seven months of anti-TB treatment initiation. Time to death was significantly associated with type of TB [Log Rank (Mantel-Cox) (p = 0.001), Breslow (Generalized Wilcoxon) (p = 0.01) and Tarone-Ware (p = 0.003) (Additional file 1: Figure S1)], antiretroviral therapy [Log Rank (Mantel-Cox) (p = 0.01), Breslow (Generalized Wilcoxon) (p = 0.024) and Tarone-Ware (p = 0.015) (Additional file 1: Figure S2)] and cotrimoxazole prophylaxis therapy [Log Rank (Mantel-Cox) (p = 0.04), Breslow (Generalized Wilcoxon) (p = 0.125) and Tarone-Ware (p = 0.08) (Additional file 1: Figure S3)].

#### Predictors of mortality

Type of TB, ART and CPT of the HIV patients were significantly associated with time to death (p < 0.05). On multivariate analysis type of TB and CPT were found to be the predictors of time to death. Patients diagnosed with extra-pulmonary TB (AHR = 17.38, 95% CI; 3.88–77.86, p < 0.001) were 17 times more likely to die than patients with pulmonary TB. Moreover, TB/HIV co-infected patients who were receiving CPT (AHR = 0.15, 95% CI; 0.03–0.74, p = 0.02) were 85% less likely to die than patients who were not on CPT (Table 2).

**Table 2:** Predictors of mortality among TB patients in Mekelle city, Northern Ethiopia, 2012–2016

Variables	Death status		AHR	95% CI	p-value
	Alive	Died			
Type of TB					
Pulmonary TB	300 (93.5)	21 (6.5)	1	1	1
EPTB	382 (85.3)	66 (14.7)	17.376	3.88–77.86	< 0.001*
ART status for TB/HIV coinfection					
ART non user	62 (75.6)	20 (24.4)	1	1	1
ART user	176 (91.2)	17 (8.8)	1.663	0.542–5.1	0.374
CPT					
No	52 (71.2)	21 (28.8)	1	1	1
Yes	204 (90.7)	21 (9.3)	0.15	0.03–0.74	0.02*

*AHR* adjusted hazard ratio, CI confidence interval, *ART* antiretroviral therapy, *CPT* cotrimoxazole prophylaxis therapy, *TB* tuberculosis, *EPTB* extra-pulmonary tuberculosis

* Statistical significance at p < 0.05

### Discussion

Of the total studied patients, 11.3% of them died over the entire follow up period. The mortality in this study was higher than findings reported in Addis Abeba [9, 10], Gonder (10%), Ethiopia [11], Brazil (10.5%) [12], Nigeria (9.9%) [13] and the national mortality in Ethiopia [14], but lower than the mortality in Vaud country, Switzerland (14%) [15] and Zimbabwe (22%) [16]. The relatively higher death rates in the present study might be due to the difference in the study sites, by which our study was conducted in two hospitals, one comprehensive specialized hospital and one referral hospital, hence the higher death rates in hospitals might be due to hospitalization of more critical TB patients in hospitals than in health centers. Moreover, the study design, target group patients might have its own role death rate variation among the countries. For instance, the study conducted in Brazil [A2] enrolled pulmonary TB patients only. Meanwhile, the socioeconomic status of countries has also its own contribution on heterogeneity of death rate among various studies globally.

Concerning the predictors of mortality, CPT was a protective factor from mortality among individuals infected with HIV including those on ART. In line with this finding, studies from Northwest Ethiopia [6], South India [17] and Sub-Saharan Africa [18] showed high risk of mortality among patients not taking CPT. Besides, in a study conducted in Nigeria patients who were not receiving CPT were at highest risk of death [13]. CPT among the simple well tolerated and cost effective strategic interventions which improve patient’s quality of life for people living with HIV/AIDS for both those on ART and those not on ART. Moreover, among individuals infected with HIV CPT is associated with a 25–46% reduction in mortality in Sub-Saharan Africa even in areas with high antibiotic bacterial resistance [19, 20].

Previous studies demonstrated positive effect of ART on survival outcomes of TB-HIV co-infected patients and highest risk of death on HIV positive patients as compared to HIV negative patients [6, 12, 16, 21, 22]. These studies depicted low risk of death among TB-HIV co-infected patients who took ART as compared to those patients who were not on ART [6, 21]. In our study we found that, ART use was not a predictor of mortality. There are previous studies that are congruent with our finding [23]. Appropriate implementation of the HAART guideline, early detection of HIV, socio economic status of patients, drug toxicities and availability of effective HAART regimens may have their own contribution on the insignificant difference among HIV reactive and non-reactive patients. Delay in initiation of ART might have its own impact on mortality difference of patients with TB-HIV coinfection. Still now there is debate on the optimal time to initiate ART in TB-HIV co-infected patients. High pill burden, immune reconstitution inflammatory syndrome (IRIS) and pharmacologic drug interactions are the challenges to early initiate ART [24]. On the other way, patient might have disease progression and die if the ART initiation is delayed [25]. In agreement to this, delaying ART initiation until after completion of TB therapy increased mortality [26, 27].

Regarding the association between type of TB and mortality, patients with extra pulmonary TB were 17 times more likely to die than those of patients with pulmonary TB including smear positive and smear negative types. Studies from Nigeria [13] and Cameron [27] also reported highest risk of death among extra-pulmonary TB patients. Extra-pulmonary TB continues to be a persistent problem and is associated with high mortality rates, especially among HIV-infected. Disseminated disease and the presence of CNS/meningeal TB are among the common extra pulmonary TB types which are associated with poor prognosis [28]. Substantial delays for early diagnosis of active TB like lack of awareness of symptoms, lack of access to health care services, and shortages of trained clinicians and laboratory personnel to make diagnosis might contribute for the noticeable rate of mortality [29–33].

### Conclusion

Our study clearly highlights higher rates of mortality and significant risk of death among extra pulmonary TB and but low risk of death among TB-HIV co-infected on CPT. Increased momentum of health care providers’ efforts is needed to improve early diagnosis of extra pulmonary TB and implement early CPT initiation for eligible patients. This study calls for strengthening efforts in mitigating mortality due to TB.

## Limitation of the study

Our study is not free of limitations. First, the study was retrospective study hence data were extracted from patient’s medical chart and registry thus it may be subjected to selection bias. Second the analyses included all deaths irrespective of the cause of death therefore there might be misclassification of the cause of death. Despite the limitations described, this study was conducted at the two major health facilities of the city with larger sample size to elucidate on the issue.

## Additional file

**Additional file 1.** Additional figures.

## Abbreviations

ACSH: Ayder Comprehensive Hospital
AHR: adjusted hazard ration
ART: antiretroviral therapy
CI: confidence interval
CPT: cotrimoxazole prophylaxis therapy
EPTB: extra pulmonary tuberculosis
HIV: human immune virus
MH: Mekelle Hospital
PTB: pulmonary tuberculosis
SNPTB: smear negative pulmonary tuberculosis
SPPTB: smear positive pulmonary tuberculosis
TB: tuberculosis
