# Supplementary material for: Time to death and risk factors among tuberculosis patients in Northern Ethiopia
Source: BMC Res Notes. 2018 Oct 4;11:696. doi: 10.1186/s13104-018-3806-7 (PMC6172746; doi:10.1186/s13104-018-3806-7)
Supplement: Supplementary file 1 — Additional file 1. Additional figures. [file 13104_2018_3806_MOESM1_ESM.docx]

Additional results for ’’**Time to Death and Risk Factors among Tuberculosis Patients in Northern Ethiopia**’’

This additional results provides additional figures and details results of the survival curve analysis of time to TB death.

**Additional results figure**

Additional figure S1: Survival curve of pulmonary and extra pulmonary tuberculosis patients in Mekelle, Ethiopia, 2017.

Additional figure S2: Survival curve of antiretroviral therapy for TB/HIV coinfected patients in Mekelle, Ethiopia, 2017.

Additional figure S3: Survival curve of cotrimoxazole prophylaxis therapy for TB/HIV coinfected patients in Mekelle, Ethiopia, 2017.


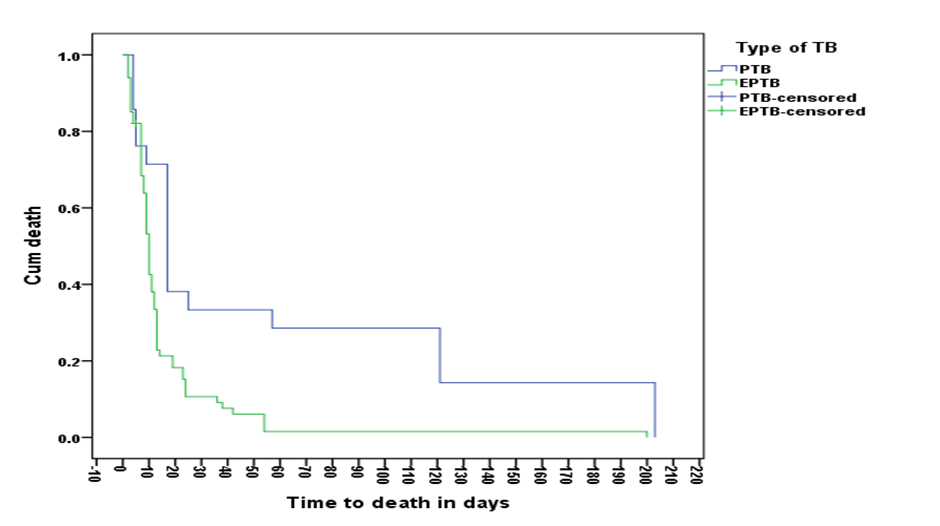


**Additional figure S1: Survival curve of pulmonary and extra pulmonary tuberculosis patients in Mekelle, Ethiopia, 2017.**


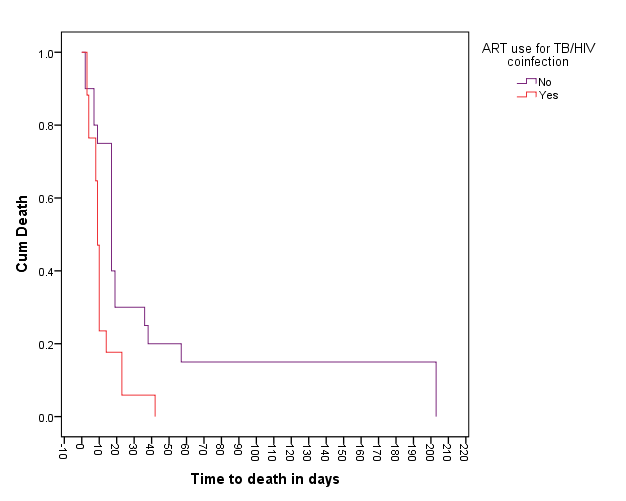
 **Additional figure S2: Survival curve of antiretroviral therapy for TB/HIV coinfected patients in Mekelle, Ethiopia, 2017.**


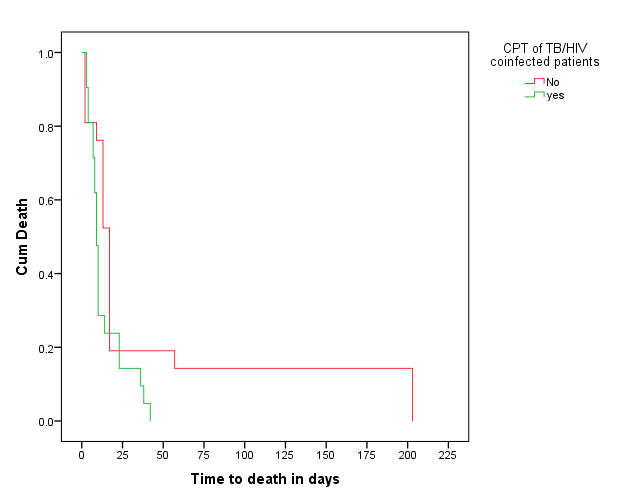
 **Additional figure S3: Survival curve of cotrimoxazole prophylaxis therapy for TB/HIV coinfected patients in Mekelle, Ethiopia, 2017.**
